# Bogus Medical Journalism

**Published:** 1896-02

**Authors:** 


### BOGUS MEDICAL JOURNALISM.

The *Medical Record* (November 16) has an excellent and timely editorial on this subject, which is so fully in accord with remarks which we have made in this paper, that we reproduce it entire:

While the *Medical Record* hails with satisfaction the advent of any new medical journal which appears to have a legitimate sphere, and always extends to it such encouragement as lies in its way, it seems right that a word of condemnation should be uttered respecting the too numerous periodicals which are launched upon the profession without any reasonable necessity or justification. Not a few are conceived solely by advertising agents—men who have neither interest nor sympathy with the profession save as they can make it a means of livelihood for themselves—and published at more or less nominal prices in anticipation of a circulation which will enable them to obtain the advertisements upon which they depend for their profits.

It is unfortunate that there are always medical gentlemen to be found who, for a small sum, are willing to "run" these journals. Again, it not infrequently happens that a medical journal is started by some doctor who is desirious of exploiting his own abilities and skill, and at the same time add to his library such books as he may be able to induce publishers to send to it for "review." We know of journals published in this country, by individuals and by associations of physicians, whose almost sole *raison d'être* are the exchanges and editorial copies of books which they obtain, without which they would cease to exist, and to obtain which they must always speak of them in terms of commendation. The publishers, not the subscribers, must be considered first. Medical journals of their class are a delusion and a snare to the profession, and an injury to all rightly conceived and conducted periodicals.

As we have often stated, we believe in local medical journals and their generous support by their legitimate constituency; and we cannot but regret that they should have to compete with journals such as we have mentioned. Of course, there is no way to prevent the publication of these illegitimate journals, and the only recourse in the hands of the profession is, as far as possible, to patronize only those of whose origin and management they have some personal knowledge. In other words, avoid subscribing to medical journals which have not good and well-known pedigrees on the sides of both publisher and editor.

